# Polysaccharides from *Artocarpus heterophyllus* Lam. (jackfruit) pulp improves intestinal barrier functions of high fat diet-induced obese rats

**DOI:** 10.3389/fnut.2022.1035619

**Published:** 2022-11-03

**Authors:** Shunjiang Zeng, Jun Cao, Yuzi Chen, Chuan Li, Gang Wu, Kexue Zhu, Xiaoai Chen, Fei Xu, Qibing Liu, Lehe Tan

**Affiliations:** ^1^Spice and Beverage Research Institute, Chinese Academy of Tropical Agricultural Sciences, Wanning, China; ^2^College of Food Science and Engineering, Hainan University, Haikou, China; ^3^College of Food Science and Technology, Huazhong Agricultural University, Wuhan, China; ^4^Key Laboratory of Processing Suitability and Quality Control of the Special Tropical Crops of Hainan Province, Wanning, China; ^5^Department of Pharmacology, School of Basic Medicine and Life Science, Hainan Medical University, Haikou, China

**Keywords:** *Artocarpus heterophyllus* Lam. polysaccharide, intestinal function, inflammation, protective effect, obese rats

## Abstract

Polysaccharides show protective effects on intestinal barrier function due to their effectiveness in mitigating oxidative damage, inflammation and probiotic effects. Little has been known about the effects of polysaccharides from *Artocarpus heterophyllus* Lam. pulp (jackfruit, JFP-Ps) on intestinal barrier function. This study aimed to investigate the effects of JFP-Ps on intestinal barrier function in high fat diet-induced obese rats. H&E staining and biochemical analysis were performed to measure the pathological and inflammatory state of the intestine as well as oxidative damage. Expression of the genes and proteins associated with intestinal health and inflammation were analyzed by RT-qPCR and western blots. Results showed that JFP-Ps promoted bowel movements and modified intestinal physiochemical environment by lowering fecal pH and increasing fecal water content. JFP-Ps also alleviated oxidative damage of the colon, relieved intestinal colonic inflammation, and regulated blood glucose transport in the small intestine. In addition, JFP-Ps modified intestinal physiological status through repairing intestinal mucosal damage and increasing the thickness of the mucus layer. Furthermore, JFP-Ps downregulated the inflammatory genes (TNF-α, IL-6) and up-regulated the free fatty acid receptors (GPR41 and GPR43) and tight junction protein (occludin). These results revealed that JFP-Ps showed a protective effect on intestinal function through enhancing the biological, mucosal, immune and mechanical barrier functions of the intestine, and activating SCFAs-GPR41/GPR43 related signaling pathways. JFP-Ps may be used as a promising phytochemical to improve human intestinal health.

## Introduction

The intestine is the largest digestive and immune organ which closely linked to the body health, participates in the digestion and absorption of nutrients. The intestine also provides a favorable anaerobic environment for microbial colonization, plays an important role in defending the host from pathogens, and regulating the host’s immune system (1–4). The contents of short-chain fatty acids (SCFAs) and colonic water, and colonic pH value are basic indicators for intestinal health, which is closely linked to the body health (5). Changes in dietary composition can affect the development of gastrointestinal disease and the integrity of the gut by altering the growth and metabolism of the intestinal flora (6). Wang et al. (7) found that walnut green husk polysaccharides alleviated obesity, chronic inflammatory responses, non-alcoholic fatty liver disease and colonic tissue damage *via* regulating gut microbiota and SCFAs content.

Polysaccharides have been reported to improve gut health *via* enhancing intestinal barrier function and restoring intestinal homeostasis, and regulate intestinal function *via* mitigating oxidative damage and inflammation, and probiotic function (8, 9). Polysaccharides from *Cyclocarya paliurus* leaves, *Dendrobium officinale*, fruiting body of *Hericium erinaceus* and polysaccharide-rich sage weed extracts were found to maintain intestinal health through lowering fecal pH value, increasing fecal water content and SCFAs concentration (5, 6, 10, 11). Gao et al. (12) reported that polysaccharide fractions from okra improved intestinal function *via* increasing the contents of SCFAs and caecum moisture, thickness of mucosa and muscular layer. *Dendrobium huoshanense* polysaccharide improved intestinal mucosal barrier function by modifying intestinal mucosal structures, regulating the production of cytokines and promoting the expression of the tight junction proteins (8).

Polysaccharides from *Artocarpus heterophyllus* Lam (jackfruit) pulp (JFP-Ps) has been demonstrated to possess immunomodulatory effects (13). In the past few years, our team has investigated the isolation, purification, *in vitro* digestive properties, antioxidant activity and *in vivo* prebiotic effects of JFP-Ps (14–16). However, to our knowledge, little has been known about the protective effects of JFP-Ps on intestinal health. Therefore, the present study aimed to investigate the protective effects of JFP-Ps on intestinal function of obese rats induced by a high-fat diet.

## Materials and methods

### Materials and reagents

Jackfruit fruits were collected from Xinglong Tropical Botanical Garden (Wanning, Hainan, China). JFP-Ps was extracted from the *Artocarpus heterophyllus* Lam pulp using hot water extraction and ethanol precipitation as previously described by Zhu et al. (14). JFP-Ps was mainly composed of Rha, Ara, Gal, Glc, Xyl, and GalA, with an average molecular weight of approximately 1,668 kDa.

The normal-chow diet (D12450H, 10% calories from fat) and high-fat diet (D12451, 45% calories from fat) were obtained from Jiangsu Synergy Pharmaceutical and Biological Engineering Co., Ltd., (Jiangsu, China). Assay kits for the activity of superoxide dismutase (SOD), glutathione peroxidase (GSH-Px) and catalase (CAT) and the content of malondialdehyde (MDA) were obtained from Suzhou Grace Biotechnology Co., Ltd., (Jiangsu, China). ELISA kits for myeloperoxidase (MPO), tumor necrosis factor-α (TNF-α), interleukin-1β (IL-1β), interleukin-6 (IL-6), interleukin-10 (IL-10) and sodium-glucose cotransporter 1 (SGLT1) were purchased from Shanghai Enzyme-linked Biotechnology Co., Ltd., (Shanghai, China). The qPCR primers for TNF-α, IL-6, G protein-coupled receptor 43 (GPR43), G protein-coupled receptor 41 (GPR41) and β-actin were purchased from Sangon Biotech (Shanghai) Co., Ltd., (Shanghai, China). TriQuick reagent and bicinchoninic acid (BCA) assay kits were purchased from Beijing Solarbio Science & Technology Co., Ltd., (Beijing, China). SuperReal Premix Plus assay kits were purchased from Tiangen Biotech (Beijing) Co., Ltd., (Beijing, China). Rabbit-derived polyclonal antibodies against occludin (27260-1-AP) and secondary antibody (SA00001-2) were purchased from Proteintech Group, Inc., (Wuhan Sanying, Hubei, China). BeyoRT*™* III first-strand synthesized kit, BeyoECL Plus reagent, poly (vinylidene fluoride) (PVDF) membrane, rabbit-derived monoclonal antibodies against β-actin (AF5003) and paraformaldehyde were purchased from Beyotime Biotechnology (Shanghai, China).

### Animal experiments

Sprague-Dawley rats (SPF-grade, male), with body weights (BW) ranging from 180 to 200 g, were purchased from Hunan Silaikejingda Experimental Animal Co., Ltd., (Changsha, China) with the experimental animal production license SCXK (Xiang) 2019-0004. All rats were housed in an animal facility under controlled interior temperature (23 ± 2°C), relative humidity (55 ± 15%), noise (≤60 dB) and lighting cycle (12:12 h light-dark). After 1-week acclimation, the rats were divided into two groups: normal control (NC) group (*n* = 8) fed with normal-chow diet, obesity group (*n* = 45) fed with high fat diet (HFD). After 12 weeks, the average body weight (BW) of the obesity group was significantly higher than that in the normal group (*p* < 0.01). The animals in obesity group were further randomly divided into five groups: HFD group, inulin group, low-, medium- and high- dose JFP-Ps groups (JFP-Ps-L, JFP-Ps-M, JFP-Ps-H), and continually fed with the high fat diet. During the diet intervention, the NC group and HFD group were treated daily with an equivalent volume of distilled water by oral gavage. The inulin group was treated daily with inulin (1.5 g/kg BW) by oral gavage (17, 18). The JFP-Ps-L group, JFP-Ps-M group and JFP-Ps-H group were treated daily with 50, 100, and 200 mg/kg JFP-Ps by oral gavage, respectively. The diet intervention was lasted for 6 weeks. The recipes of the diets are listed in S1 and S2. All animal experimental procedures were approved by the Animal Ethics Committee of Hainan University and Hainan Medical University with experimental animal use permit SYXK (Qiong) 2017-0013.

### Sample collection

All the rats were fasted for 12 h and anesthetized with chloral hydrate by intraperitoneal injection, and then dissected. Fecal samples were collected, immersed immediately in liquid nitrogen, and stored at −80°C for water content and pH value analysis. The length of the colon was measured and the intestinal tissue was rinsed with pre-cooled saline, blotted on filter paper, and divided into three portions for further analysis.

### Water content and pH value of feces

A portion of fecal sample was heated in an air-oven at 105 ± 2°C to a constant weight. The water content of the feces was calculated from the mass of the feces before and after drying. The other part of fecal sample was diluted with distilled water at a ratio of 1:10 (w/v) and the pH value was determined using a SevenCompact*™* S220 pH meter (Mettler Toledo, Switzerland).

### Antioxidant activities analysis

One hundred milligram colon tissue were mixed with pre-cooled saline (4°C, 0.9%, w/w) at a ratio of 1:10 (w/v), homogenized over ice for 3 min, and centrifuged (12,000 × g, 15 min, 4°C) to gather supernatants. The activities of SOD, GSH-Px and CAT and the content of MDA were determined using the biochemical kits following the manufacturer’s protocols.

### Enzyme-linked immunoassay

One hundred milligram of colon tissue were homogenized with 1.0 mL pre-cooled saline (4°C, 0.9%, w/w) over ice for 5 min. Then tissue homogenate was centrifuged (12,000 × g, 20 min, 4°C) to collect supernatants. The activity of MPO and the concentrations of TNF-α, IL-1β, IL-6, and IL-10 in the colon were measured by ELISA assay kits following the manufacturer’s instructions. The activity of SGLT1 in the small intestine was analyzed following the manufacturer’s instructions.

### Histological examination

The small intestine tissue was fixed in 4% paraformaldehyde overnight, dehydrated with graded alcohol, imbedded in a paraffin wax, and whittled down to 4 μm thickness. Then the sections were mounted onto clean glass slides, soaked in graded xylene and alcohol, stained with hematoxylin and eosin. Lastly, slides were sealed with neutral balsam for inspection under a binocular microscope.

### RT-qPCR analysis

The mRNA levels of TNF-α, IL-6, GPR43, and GPR41 were determined by RT-qPCR. Total RNA was extracted from the small intestine tissue with the TriQuick reagent according to the manufacturer’s protocol. The purity and concentration of total RNA was determined by Thermo Scientific*™* NanoDrop*™* 2000C spectrophotometer. cDNA was generated by reverse transcription of RNA using BeyoRT*™* III first-strand synthesized kit. RT-qPCR analysis of the target genes was performed on the Bio-Rad^®^ CFX96 Real Time PCR System using a SuperReal PreMix Color (SYBR Green). The relative expression levels of the genes were calculated according to the 2^–ΔΔ Ct^ method and normalized to the housekeeping gene, β-actin. Primer information is listed in Table 1.

**TABLE 1:** The primer sequences for amplification in RT-qPCR.

Target gene	Primer	Sequence (5′–3′)	Product size (bp)
β-actin	Forward	TGTCACCAACTGGGACGATA	165
	Reverse	GGGGTGTTGAAGGTCTCAAA	
TNF-α	Forward	AAAGGACACCATGAGCACGGAAAG	136
	Reverse	CGCCACGAGCAGGAATGAGAAG	
IL-6	Forward	ACTTCCAGCCAGTTGCCTTCTTG	110
	Reverse	TGGTCTGTTGTGGGTGGTATCCTC	
GPR43	Forward	TGCACCATCGTCATCATCGTTCAG	137
	Reverse	ACCAGGCACAGCTCCAGTCG	
GPR41	Forward	TCTGCTCCTCTTCCTGCCATTCC	150
	Reverse	CGTTCTATGCTCACCGTCATCAGG	

### Western blot analysis

Fifty milligram of small intestine tissue were homogenized with 0.5 mL pre-cooled RIPA buffer, 5 μL PMSF lysis buffer and 5 μL protease inhibitor cocktail over ice for 5 min, and centrifuged (12,000 × g, 10 min, 4°C) to collect supernatants. The protein concentrations were quantified by BCA Protein Assay Kit. Denatured protein samples were fractionated on a 10% SDS-PAGE and transferred onto 0.45 μm PVDF membranes. After the membranes were blocked with 5% skimmed milk at room temperature for 60 min, the membranes were incubated overnight with primary antibodies (1:1,300) at 4°C, and then HRP-conjugated secondary antibody (1:1,000) following the manufacturer’s instructions. Protein bands were developed using an ultrasensitive ECL chemiluminescence kit and visualized using a Tanon 5200 Multi chemiluminescent imaging system, and lastly quantified using Image J software.

### Statistical analysis

Results are expressed as means ± standard error of the mean (SEM). Data were analyzed by one-way analysis of variance (ANOVA), followed by Duncan’s multiple comparison tests with SPSS Statistics 26 software (IBM, USA). *p* < 0.05 indicated a statistically significant difference.

## Results

### Effects of JFP-Ps on water content and pH value of feces

**FIGURE 1:**
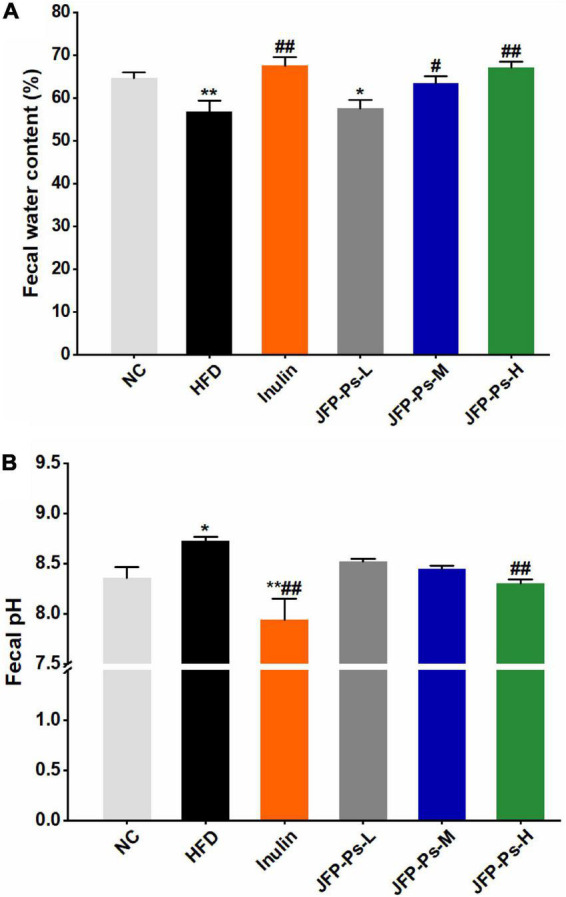
Effect of JFP-Ps on fecal water content **(A)** and fecal pH value **(B)** in obese rats. Data are expressed as mean ± SEM (*n* = 6 for each group) and analyzed using one-way ANOVA. *p < 0.05, **p < 0.01 compared to the NC group; ^#^*p* < 0.05, ^ ##^*p* < 0.01 compared with the HFD group.

The water content of feces was significantly lower in the HFD group (56.75 ± 2.64%) than that in the NC group (64.68 ± 1.28%) (Figure 1A). JFP-Ps and inulin treatments increased water content as compared with HFD treatment. Moreover, the water content in the JFP-Ps-H group (67.05 ± 1.34%) was close to that in the inulin group (67.47 ± 2.02%), which was slightly higher than that in the normal group. As shown in Figure 1B, the pH value of feces in the HFD group was significantly higher than the NC group (*p* < 0.05). After feeding with JFP-Ps and inulin, the pH values of feces were significantly decreased in obese rats compared with the HFD treatment. The results indicated that JFP-Ps significantly increased fecal water content and decreased fecal pH value in obese rats.

### Effects of JFP-Ps on colon length and intestinal micromorphology

**FIGURE 2:**
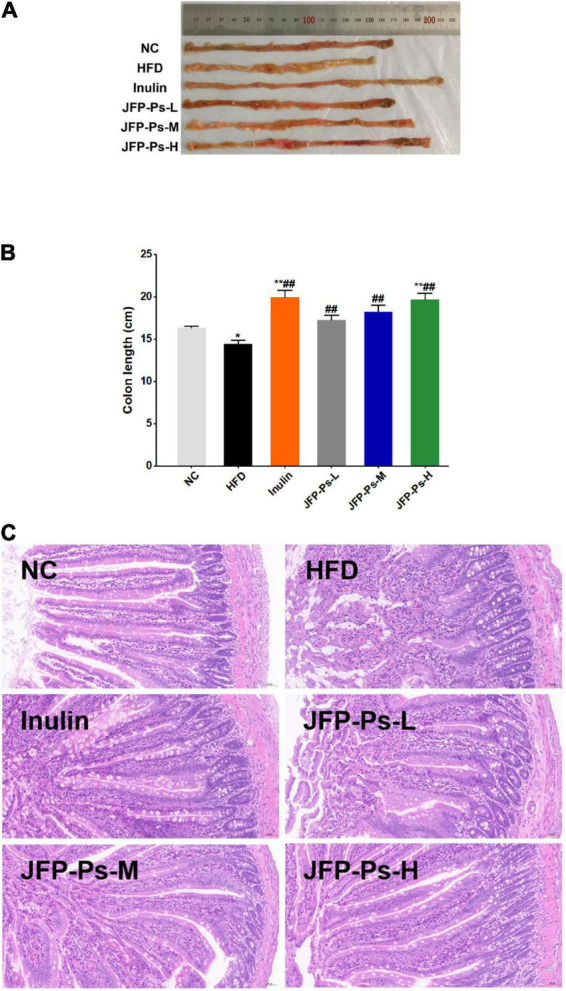
Effect of JFP-Ps on colon length and intestinal micromorphology in obese rats. **(A)** Representatives of colonic tissue in each group, **(B)** colon length, **(C)** jejunal micromorphology (original magnification 20X). Data are expressed as mean ± SEM (*n* = 6 for each group) and analyzed using one-way ANOVA. *p < 0.05, **p < 0.01 compared to the NC group; ^##^*p* < 0.01 compared with the HFD group.

As shown in Figures 2A,B, the colon of the HFD group was significantly shorter than the NC group (*p* < 0.05). JFP-Ps and inulin significantly inhibited the decrease of colon length compared with the HFD group (*p* < 0.01).

Morphological changes in jejunal tissue between the groups were visualized by H&E staining. As shown in Figure 2C, the jejunum in the NC group showed clear tissue structure, with finger-shaped villi closely and evenly arranged, a large number of cup-shaped cells within the columnar epithelium, and a clear demarcation between the submucosa and the muscular layer. In the HFD group, the mucosal layer of jejunum was disorganized; the shape of the villi was not clear; and the tips of the villi were mixed and accompanied by severe inflammatory infiltration, with cup-shaped cells visible only at the base of the villi. Comparison to the HFD group, the jejunum of the JFP-Ps-L group and JFP-Ps-M group showed clearer structure, with the villi gradually becoming clearer in shape and more uniformly arranged, with less mixing of the villi apices and less inflammatory infiltration, and a gradual increase in the distribution of cupped cells within the columnar epithelium. The morphology and structure of the jejunal tissue in the JFP-Ps-H group was similar to that in the NC group, but the length and number of villi increased. Moreover, villi were more closely arranged; the volume of the intestinal lumen decreased; more cup-shaped cells were distributed within the columnar epithelium and the thickness of the mucosal layer increased. The results showed that JFP-Ps restored intestinal mucosal damage induced by a high-fat diet in obese rats, increased the thickness of the mucus layer and protected the barrier function of the intestinal mucosa, which in turn had a positive effect on intestinal health.

### Effects of JFP-Ps on antioxidant activities in colon

As shown in Table 2, in comparison to the NC group, the activities of SOD, GSH-Px, and CAT in the HFD group were decreased and the content of MDA in the HFD group was increased (*p* < 0.01). Compared with HFD group, JFP-Ps treatment increased the activities of SOD, GSH-Px and CAT, and the activities of GSH-Px increased significantly (*p* < 0.01). Moreover, JFP-Ps decreased the content of MDA significantly (*p* < 0.01). The results indicated that JFP-Ps may alleviate oxidative damage in the colon of obese rats and improve integrity of the intestinal epithelium by enhancing the activities of antioxidant enzymes in the colon.

**TABLE 2:** Effect of JFP-Ps on antioxidant activities in the colon of obese rats.

Group	SOD (U/mL)	GSH-Px (nmol/mL)	CAT (μ mol/mL)	MDA (nmol/mL)
NC	39.64 ± 2.57	97.35 ± 3.91	117.16 ± 18.75	2.47 ± 0.17
HFD	22.72 ± 2.28	54.92 ± 2.38**	77.48 ± 2.72	3.85 ± 0.10**
Inulin	33.40 ± 1.59	109.12 ± 3.54^##^	104.28 ± 17.24	2.44 ± 0.14^##^
JFP-Ps-L	25.08 ± 9.18	96.73 ± 1.63^##^	98.42 ± 15.27	2.94 ± 0.45^#^
JFP-Ps-M	30.24 ± 8.80	108.34 ± 4.32^##^	113.46 ± 32.90	2.06 ± 0.18^##^
JFP-Ps-H	34.68 ± 3.05	105.87 ± 5.33^##^	115.53 ± 12.86	1.80 ± 0.06^##^

Data are presented as mean ± SEM (*n* = 6). **p < 0.01 compared to the NC group; ^#^*p* < 0.05, ^##^*p* < 0.01 compared with the HFD group.

### Effects of JFP-Ps on inflammation-related indicators in colon

As shown in Table 3, the MPO activity in the HFD group was significantly increased (*p* < 0.05), and the contents of pro-inflammatory cytokines (TNF-α, IL-1β, and IL-6) increased, and the content of anti-inflammatory cytokine (IL-10) decreased, compared to that in the NC group. However, compared with HFD group, the MPO activity in the JFP-Ps group was significantly decreased (*p* < 0.01), the contents of pro-inflammatory cytokines were decreased in a dose-dependent manner, and the level of the anti-inflammatory cytokine was increased. The results indicated that JFP-Ps may decrease the inflammation in the colon of obese rats induced by high-fat diet.

**TABLE 3:** Effect of JFP-Ps on inflammation-related factors in the colon of obese rats.

Group	MPO (U/L)	TNF-α (ng/L)	IL-1β (ng/L)	IL-6 (pg/mL)	IL-10 (ng/L)
NC	50.74 ± 0.71	82.25 ± 6.29	5.55 ± 0.12	29.68 ± 0.70	6.70 ± 1.53
HFD	58.10 ± 3.44*	89.87 ± 12.44	6.30 ± 0.54	36.98 ± 5.73	4.21 ± 0.33
Inulin	47.71 ± 2.11^#^	81.89 ± 1.56	4.91 ± 0.30^#^	32.12 ± 2.75	6.34 ± 0.30
JFP-Ps-L	46.67 ± 2.61^##^	83.32 ± 8.35	5.67 ± 0.12	32.39 ± 5.24	5.67 ± 0.60
JFP-Ps-M	46.63 ± 2.68^##^	73.92 ± 4.03	5.59 ± 0.41	27.88 ± 2.90	5.79 ± 0.95
JFP-Ps-H	45.70 ± 1.32^##^	72.37 ± 0.95	5.24 ± 0.37	26.44 ± 1.85	5.86 ± 0.26

Data are presented as mean ± SEM (*n* = 6). *p < 0.05 compared to the NC group; ^#^*p* < 0.05, ^ ##^*p* < 0.01 compared with the HFD group.

### Effects of JFP-Ps on glucose transport in the small intestine

**FIGURE 3:**
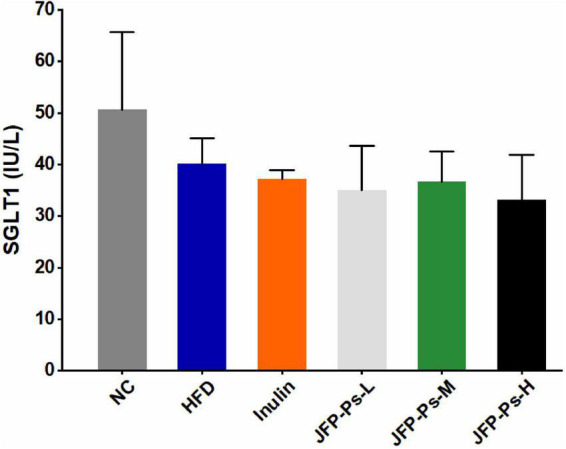
Effect of JFP-Ps on SGLT1 activity in small intestinal tissues. Data are expressed as mean ± SEM (*n* = 6 for each group) and analyzed using one-way ANOVA. No significant difference was observed.

As shown in Figure 3, SGLT1 activity in the small intestine of rats from the HFD group was decreased compared with the NC group. The JFP-Ps intervention reduced SGLT1 activity in HFD group. The results showed that JFP-Ps may inhibit glucose transport in the small intestine by reducing SGLT1 activity in the intestinal epithelium of obese rats.

### Effects of JFP-Ps on the expression of gut barrier function-related genes in small intestine

**FIGURE 4:**
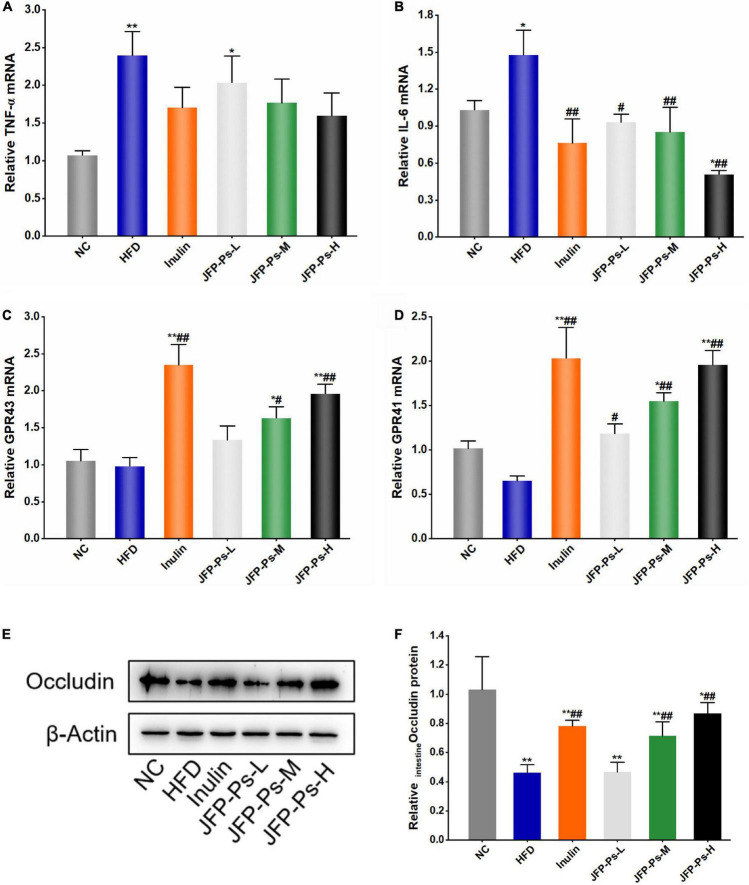
Effect of JFP-Ps on the expression of inflammatory genes, free fatty acid receptor genes and tight junction protein in the small intestine of obese rats. **(A)** TNF-α, **(B)** IL-6, **(C)** GPR43, **(D)** GPR41, **(E)** protein expression of occludin, **(F)** relative band intensities of occludin. Data are expressed as mean ± SEM (*n* = 6 for each group) and analyzed using one-way ANOVA. *p < 0.05, **p < 0.01 compared to the NC group; ^#^*p* < 0.05, ^##^*p* < 0.01 compared with the HFD group.

The levels of TNF-α and IL-6 mRNA expression were significantly increased in the small intestine of the HFD group, and the gene expression levels of GPR41 and GPR43 were slightly down-regulated (Figures 4A–D). Interestingly, treatment with JFP-Ps decreased the expression of TNF-α and IL-6, while increased the gene expression of GPR43 and GPR41 in a dose-dependent manner. These results showed that JFP-Ps may inhibit inflammation and enhance immune function in the small intestine of obese rats.

### Effects of JFP-Ps on the protein expression level of occludin in small intestine

Western blot analysis showed that protein expression level of occludin in the HFD group was significantly lower than the NC group (*p* < 0.01) (Figures 4E,F). JFP-Ps significantly increased the protein expression of occludin in a concentration-dependent manner (*p* < 0.01). The result showed that JFP-Ps may enhance mechanical barrier function in the small intestine.

## Discussion

Intestine is the largest digestive and immune organ in the body, provides a favorable and anaerobic environment for microbial colonization and performs an important role in nutrient absorption, detoxification and immune regulation (2, 3). Intestinal dysfunction is closely associated with obesity and other related metabolic diseases (19, 20). Natural polysaccharides could prevent and treat intestinal diseases caused by various factors *via* restoring intestinal barrier function (21). The results of this study indicated that JFP-Ps possessed a protective effect on the intestine *via* improving intestinal barrier function and alleviating intestinal inflammation.

The higher water content in the feces is accompanied by an increase in the volume and looseness of the feces, which facilitates the body’s bowel movements (22). A high water content in feces also indicates a high moisture environment in the intestine, which may help the exchange and transport of substances and the intestinal mucus layer in dissolving mucins, immune factors, electrolytes, etc., thus maintaining the integrity of the intestinal mucosal layer and the balance of osmotic pressure and protecting the intestinal barrier (23–25). In addition, water in the intestinal lumen enters the enterocytes in the villi by osmosis, causing the cells to swell and the tissue to thicken, leading to spontaneous bending of adjacent tissue, promoting the formation of crypt foci in the intestine, and reducing the lumen volume (26). Do et al. (27) reported that polysaccharide fraction from greens of *Raphanus sativus* increased water content in the feces of obese mice induced by high-fat diet. JFP-Ps increased the water-holding capacity of feces and promoted the body’s bowel movements, implying that the intake of JFP-Ps may contribute to the integrity of the intestinal mucosal layer, formation of the crypt, and protection of the intestinal barrier.

The pH value of the intestinal contents is an important parameter in measuring intestinal health (28). An increased intestinal pH value is associated with a decrease in the abundance of probiotic bacteria (such as *Lactobacillus* and *Bifidobacterium*) and an increase in the abundance of pathogenic bacteria. Oligosaccharides were fermented by intestinal microorganisms to produce SCFAs, lowering intestinal pH value and promoting the growth of probiotic bacteria (29). In the study, JFP-Ps was found to decrease intestinal pH value, suggesting that JFP-Ps may be fermented by intestinal microorganisms to produce SCFAs, creating an acidic intestinal environment, inhibiting the growth of harmful bacteria, promoting the growth of probiotic bacteria and the balance of intestinal flora, thereby enhancing the biological barrier function of the intestine in the obese rats. The results were consistent with our previous study (16).

The colon acts as an important site for absorbing water and salt from food residues and provides a habitat for intestinal flora. Dietary fibers are fermented in the colon, and the timing and effect of fermentation is influenced by the growth state of the colon (30). The colon length may be shortened due to intestinal diseases. However, the butyric acid produced by intestinal probiotics (e.g., *Lactobacillus, Bifidobacterium*, etc.) from the fermentation of dietary polysaccharides provides 60–70% energy for the colon cells, promoting the regeneration and growth of colon epithelial cells, which in turn increases colon length (30, 31). Polysaccharide fraction from greens of *Raphanus sativus* was reported to increase colon length and restore intestinal barrier function in high-fat diet induced obese mice (27). In the study, JFP-Ps increased colon length in a dose-dependent manner, restored intestinal mucosal damage and increased the thickness of the mucus layer in obese rats induced by a high-fat diet, suggesting that JFP-Ps may enhance intestinal barrier function and reduce the risk of intestinal diseases.

The antioxidant enzymes SOD, GSH-Px and CAT constitute the body’s enzymatic antioxidant system, play an important role in protecting the body from oxidative damage, and are regarded as the first line of defense against oxidative damage (32). MDA is a product of lipid peroxidation in tissues and organs, and its level reflects the degree of tissue damage. A high-fat diet causes oxidative damage in the body and promotes the development of obesity. Wang et al. (7) reported that walnut green husk polysaccharides could prevent colonic oxidative stress and inflammation damage caused by high-fat diets. Our previous study has found that JFP-Ps exhibited a strong free radical scavenging activity (14). Consistent with these reports, JFP-Ps increased the activities of SOD, GSH-Px and CAT and decreased the content of MDA, suggesting that JFP-Ps may maintain the integrity of intestinal epithelium by increasing the activities of antioxidant enzymes in the colon of obese rats induced by a high-fat diet.

Weakening of the intestinal mucosal barrier allows a large number of foreign antigens to enter the intestinal wall, inducing an inflammatory response in the gut and accumulation of inflammatory cells and inflammatory cytokines, and triggering an immune response and damage (33). MPO is a hemoglobin enriched in neutrophils, and MPO activity was used to reflect neutrophil aggregation and tissue inflammation. TNF-α, IL-1β and IL-6 are typical pro-inflammatory cytokines that promote the inflammatory cascade; IL-10 is an anti-inflammatory cytokine that inhibits the inflammatory response. Liu et al. (34) reported that *Rheum tanguticum* polysaccharide significantly reduced MPO activity in the colonic mucosa of rats with colitis. A polysaccharide purified from *Arctium lappa* inhibited the increase of pro-inflammatory factors in the colon of mice with colitis (35). *Angelica sinensis* polysaccharide increased IL-10 level in the colon of rats with immune colonic injury (36). Consistent with these reports, JFP-Ps decreased the contents of TNF-α, IL-1β and IL-6, and increased the content of IL-10, suggesting that JFP-Ps may alleviate colonic inflammation in high-fat diet-induced obesity rats.

SGLT1 is a key transporter mainly expressed in small intestinal tissues, which involved in glucose absorption in the intestinal lumen, and closely associated with metabolic diseases, such as obesity and diabetes (37). In obese patients with type 2 diabetes, the overexpression of SGLT1 caused an abnormal increase in blood glucose in the body (38). Inhibition of SGLT1 expression can reduce glucose absorption in small intestine, the escaped glucose transferred into the large intestine and fermented to produce SCFAs, which in turn reduces the occurrence of obesity and type 2 diabetes (37, 39). Consistent with the previous study, JFP-Ps reduced SGLT1 activity in the intestinal epithelial cells of obese rats, suggesting that JFP-Ps may alleviate the development of obesity.

The expression levels of TNF-α and IL-6 in small intestinal tissues may reflect the inflammatory status of small intestinal tissues in obese rats. Han et al. (40) found that polysaccharide from *Gracilaria Lemaneiformise* reduced intestinal inflammation and prevented colitis in mice by decreasing the levels of pro-inflammatory factors in the mouse colon. GPR41 and GPR43 is a group of free fatty acid receptors that are activated by SCFAs. Intestinal microbes can ferment indigestible dietary fiber to produce SCFAs, which in turn activate GPR41 and GPR43, and mediate immune function in the small intestine (41–43). Consistent with these reports, our results showed that JFP-Ps down-regulated the expression of pro-inflammatory genes (TNF-α, IL-6), up-regulated the expression of GPR41 and GPR43. These results suggested that JFP-Ps could alleviate inflammation and enhance immune barrier function of the small intestine in obese rats *via* inhibiting the expression of pro-inflammatory genes and activating SCFA- and GPR41/GPR43-related signaling pathways in the small intestine of obesity rats.

Occludin is a tight junction protein and is believed to be directly involved in the barrier and fence functions of tight junctions (44). Occludin has been identified as an important component of the intestinal mechanical barrier and regulates macromolecule flux across the intestinal epithelial tight junction barrier (45). Sang et al. (46) reported that polysaccharide from sporoderm-broken spore of *Ganoderma lucidum* up-regulated the expression of occludin protein in the ileum of mice fed with high-fat diet. In line with this report, JFP-Ps increased the protein expression of occludin in a concentration-dependent manner, suggesting that JFP-Ps may enhance mechanical barrier function in the small intestine of obese rats.

## Conclusion

**FIGURE 5:**
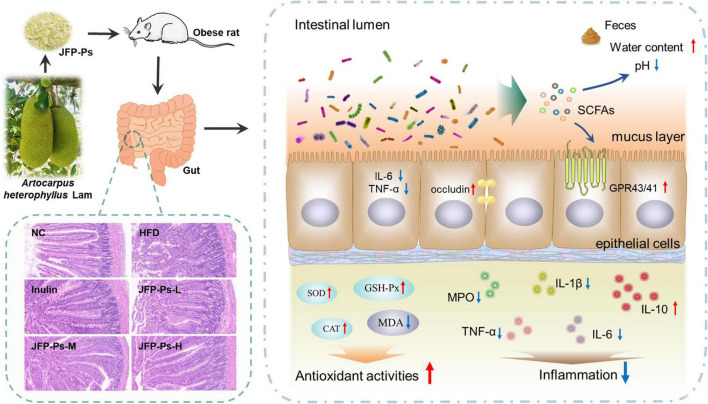
The beneficial effects of JFP-Ps on intestinal barrier function in high fat diet-induced obese rats.

In conclusion, JFP-Ps exhibited a protective effect on intestinal function and was beneficial to intestinal health. As shown in Figure 5, JFP-Ps promoted bowel movements and modified intestinal physiochemical environment by lowering fecal pH value and increasing fecal water content. Meanwhile, JFP-Ps was found to increase the length of the colon, alleviate oxidative damage of the colon, relieve intestinal colonic inflammation, and inhibit glucose transport in the small intestine. In addition, JFP-Ps repaired intestinal mucosal damage, and increased the thickness of the mucus layer. The potential mechanism of JFP-Ps improved intestinal barrier functions involved in inhibiting the expression of the inflammatory genes (TNF-α, IL-6), promoting the expression of the tight junction protein (occludin), and activating SCFA-GPR41/GPR43 related signaling pathways. Our findings would provide theoretical support for the development of JFP-Ps as a promising phytochemical to improve human health.

## Data availability statement

The data presented in this study are included in the article/Supplementary material, further inquiries can be directed to the corresponding authors.

## Ethics statement

This animal study was approved by the Animal Ethics Committee of Hainan Medical University.

## Author contributions

SZ, JC, KZ, QL, and LT designed the experiments. SZ and YC conducted the experiments. CL, GW, XC, and FX analyzed the data. SZ, JC, KZ, and LT wrote and revised the manuscript and gave the final approval. All authors contributed to the article and approved the submitted version.

## Abbreviations

JFP-Ps: polysaccharides from *Artocarpus heterophyllus* Lam. (jackfruit) pulp
SOD: superoxide dismutase
GSH-Px: glutathione peroxidase
CAT: catalase
MDA: malondialdehyde
MPO: myeloperoxidase
TNF-α: tumor necrosis factor-α
IL-1β: interleukin-1β
IL-6: interleukin-6
IL-10: interleukin-10
SGLT1: sodium-glucose cotransporter 1
GPR43: G protein-coupled receptor 43
GPR41: G protein-coupled receptor 41
SCFAs: short-chain fatty acids.
