# Supplementary material for: Polysaccharides from Artocarpus heterophyllus Lam. (jackfruit) pulp improves intestinal barrier functions of high fat diet-induced obese rats
Source: Front Nutr. 2022 Nov 3;9:1035619. doi: 10.3389/fnut.2022.1035619 (PMC9669604; doi:10.3389/fnut.2022.1035619)
Supplement: Supplementary file 1 [file Data_Sheet_1.docx]

**Supplementary material**

**Table S1**

The comparison of feed formulation data

| Nutriment | D12451 | | D12450H | |
| --- | --- | --- | --- | --- |
|  | g (%) | kcal (%) | g (%) | kcal (%) |
| Protein | 23.7 | 20.1 | 19.2 | 20.1 |
| Fat | 23.6 | 45.1 | 4.3 | 10.0 |
| Carbohydrate | 40.9 | 34.7 | 66.8 | 69.9 |
| Total | - | 100 | - | 100 |
| kcal/g | 4.70 | - | 3.83 | - |

**Table S2**

The feed formulation used in this study

| Ingredient | D12451 | | D12450H | |
| --- | --- | --- | --- | --- |
|  | g (%) | kcal (%) | g (%) | kcal (%) |
| Casein | 200 | 800 | 200 | 800 |
| L-Cystine | 3 | 12 | 3 | 12 |
| Corn starch | 72.8 | 291.2 | 452.2 | 1808.8 |
| Maltodextrin | 100 | 400 | 75 | 300 |
| Sucrose | 176.8 | 707.2 | 176.8 | 707.2 |
| Cellulose | 50 | 0 | 50 | 0 |
| Soybean oil | 25 | 225 | 25 | 225 |
| Lard | 177.5 | 1597.5 | 20 | 180 |
| Mineral mix S10026B | 50 | 0 | 50 | 0 |
| Vitamin mix V10001C | 1 | 4 | 1 | 4 |
| Choline bitartrate | 2 | 0 | 2 | 0 |
| FD&C yellow dye #5 | 0 | 0 | 0.04 | 0 |
| FD&C red dye #40 | 0 | 0 | 0.01 | 0 |
| FD&C blue dye #1 | 0.05 | 0 | 0 | 0 |
| Total | 858.15 | 4036.9 | 1055.05 | 4037 |
